# An ERG and OCT study of neuronal ceroid lipofuscinosis CLN2 Battens retinopathy

**DOI:** 10.1038/s41433-021-01594-y

**Published:** 2021-07-16

**Authors:** Dorothy A. Thompson, Siân E. Handley, Robert H. Henderson, Oliver R. Marmoy, Paul Gissen

**Affiliations:** 1 Clinical and Academic Department of Ophthalmology, Great Ormond Street Hospital for Children, London, UK; 2 UCL Great Ormond Street Institute of Child Health, London, UK; 3 Genetics and Genomic Medicine, Great Ormond Street Institute of Child Health, University College London, London, UK; 4 NIHR Great Ormond Street Hospital Biomedical Research Centre, University College London, London, UK

**Keywords:** Retina, Neuroscience

## Abstract

**Background:**

Late infantile neuronal ceroid lipofuscinosis (CLN2 Batten disease) is a rare, progressive neurodegenerative disease of childhood. The natural history of motor and language regression is used to monitor the efficacy of CNS treatments. Less is known about CLN2 retinopathy. Our aim is to elaborate the nature, age of onset, and symmetry of CLN2 retinopathy using visual electrophysiology and ophthalmic imaging.

**Subjects and methods:**

We reviewed 22 patients with genetically confirmed CLN2 disease; seventeen showing classical and five atypical disease. Flash electroretinograms (ERGs), flash and pattern reversal visual evoked potentials (VEPs), recorded from awake children were collated. Available fundus images were graded, optical coherence tomography (OCT) central subfoveal thickness (CST) measured, and genotype, age, clinical vision assessment and motor language grades assembled.

**Results:**

ERGs show cone/rod system dysfunction preceded by localised macular ellipsoid zone disruption on OCT from 4.8 years. Electroencephalogram (EEG) time-locked spikes confounded both pattern 6/17 (35%) and flash VEPs 12/16 (75%). Paired right eye (RE) and left eye (LE) ERG amplitudes did not differ significantly for each flash stimulus at the *p* 0.001 level, Wilcoxon ranked signed test. Cone ERGs show a functional deficit before CST thinning in classical disease. Optomap hyper fundus autofluorescence (FAF) at the fovea was noted in three patients with normal ERGs. The oldest patient showed an ovoid aggregate above the external limiting membrane at the fovea, which did not affect the PERG.

**Conclusion:**

ERG findings in CLN2 retinopathy show symmetrical cone-rod dysfunction, from 4y10m in this series, but a broad range of ages when ERG function is preserved.

## Background

Batten disease is a collective description for all forms of neuronal ceroid lipofuscinosis (NCL) [1, 2]. NCL disorders are rare, autosomal recessive, progressive neurodegenerative lysosomal storage diseases of the brain and, in most cases, the retina. NCL diseases typically begin in childhood and arise from mutations in one of 13 known genes that produce soluble lysosomal or transmembrane proteins [3]. Patients with Batten disease have visual impairment leading to blindness, cognitive and motor decline, seizures and premature death. The symptoms occur over different time courses according to the NCL subtype. Although rare, with estimates of around 2 per 100,000, the NCLs are the most common of childhood neurodegenerative disorders [4].

Historically NCLs were grouped according to the age of onset into congenital, infantile (INCL), late infantile (LINCL), juvenile (JNCL) and adult-onset forms, or by eponymous names such as Santavuori-Haltia disease for INCL (onset 6–24 months) or Vogt–Spielmeyer–Sjogren disease for JNCL onset (5–15 year). Now NCLs are described by numerical genotype. The most common types are CLN1 (INCL), CLN2 (LINCL), CLN6 (variant LINCL) and CLN3 (JNCL) [1, 3]. Patients with CLN3 JNCL often present first to an ophthalmologist with visual loss due to maculopathy [5, 6].

Classical CLN2 LINCL or Jansky–Bielschowsky disease results from the depleted expression of the lysosomal enzyme tripeptidyl peptidase 1 (TTP1) caused by mutations in the CLN2 gene on chromosome 11p15 [7]. Children typically present between 2 and 4 years, with seizures, language and motor dysfunction and progress rapidly to develop visual loss, myoclonus and photosensitivity, deterioration of cognitive skills and death in early adolescence, [4]. Around 20% of patients with CLN2 have a “non-classical” also called “atypical” form of the disease with variable ages of presentation ([8–10] and personal communications). Whilst some non-classical patients display all the known features of LINCL progressing slowly, others lack seizures and/or retinal disease.

Clinical management is palliative, but promising treatments are being developed and evaluated [11]. Enzyme replacement therapy (ERT) is one of these [12–15]. The soluble lysosomal proteins enzymes (CLN1 (PPT1), CLN2 (TPP1), CLN10 (CTSD) and CLN13 (cathepsin F; CTSF)) can be exchanged between cells and therefore can be applied exogenously to deficient cells. ERT introduces purified recombinant enzymes via intravenous, intracerebroventricular or intrathecal injection into the subarachnoid space to bypass the blood-brain barrier [4, 11, 12]. There have been encouraging reports of treatments in CLN2 slowing decline [16–18]. ERT administration does not alter the progressive retinal degeneration [18], probably because of the spectrum of the eye’s immune privilege and blood-retina CNS barrier [19], but intravitreal administration of rhTPP1 has slowed retinal degeneration in four *TPP1*-null dogs [20]. The advent of treatments for CLN2 disease highlights an urgent need to better characterise the natural history of CLN2 retinopathy, which is shielded from some treatments by the blood-retina barrier.

The natural history of rare diseases can be used to monitor the efficacy of treatment when control groups or randomisation studies are not feasible or ethical. Natural clinical history scales for CLN2 have been developed [21–23]. The two domain motor and language CLN2 Disease Clinical Rating Scale has shown uniform and predictable progression for independently rated patients in different countries [24]. It is being used to monitor disease trajectory in the intraventricular Cerliponase Alfa study [17]. An ophthalmic severity score based on fundus appearance [25] has superseded a behavioural vision scale [21]. Structural ocular findings are typified by a gradually progressive central to peripheral retinal degeneration, starting in a bull’s eye pattern at the level of the outer retina [25, 26], but the need for functional vision outcomes such as electroretinograms (ERGs) has been highlighted [26]. CLN1 INCL and CLN3 JNCL are characterised by an extinguished or reduced rod specific ERG and an electronegative mixed rod-cone ERG. These features indicate rod system inner retinal dysfunction [5, 27, 28]. In contrast, the fundal changes described in CLN2 LINCL retinopathy anticipate the ERGs will reflect a maculopathy and progressive cone-rod dystrophy [21, 26, 27].

Our study is a retrospective case series of patients with genetically confirmed CLN2 at Great Ormond Street Hospital for Children (GOSH) that seeks to contribute functional ERG and visual evoked potential (VEP) measures, and structural imaging data to the natural history of CLN2 retinopathy.

## Patients and methods

The records of patients biallelic for mutations associated with CLN2 were reviewed. Visual electrophysiology tests, flash skin ERG, pattern VEP and flash VEP were carried out in awake children using techniques developed at GOSH. In brief, skin electrodes are positioned on the cheek below the eye and referenced to the outer canthus. Flashes are presented 3/s using a handheld Grass (Gr) strobe at different settings to produce, in the dark, a predominantly rod driven ERG to dim blue Gr1 (3/s), a maximal flash a-wave to white Gr16 (1/20 s), a mixed rod-cone ERG to white Gr4 (3/s) and in the light, a cone ERG to white Gr4 (3/s) and a flicker ERG to Gr4 (30/s). The skin ERGs produce similar shapes and have broad physiological equivalence to ISCEV ERGs, [29, 30]. The amplitudes and peak times of the ERG a- and b-waves produced to each flash stimulus were measured. To facilitate comparison with other published ERGs produced by different recording techniques we graded the amplitudes by dividing the recorded ERG by the reference limit fifth centile amplitude. Values above 1 are in the normal range, below 1 are subnormal and 1 is borderline. Inter-ocular values for ERG amplitudes were compared using the Wilcoxon signed-rank test. One patient was examined during sedation for ERT infusion using a RetEVAL portable ERG device.

Pattern and flash VEPs were recorded to ISCEV standards with both eyes open [31]. A range of high contrast check widths (400′–6.25′) reversed at 3/s in a 30° field viewed at 1 m. Flash VEPs were produced to the Gr4 stimulus and recorded simultaneously when possible with the flash skin ERG. The main positive peaks of the VEPs were measured from the Oz-Fz channel. A pattern ERG was recorded in an awake and compliant patient to ISCEV PERG standards [32].

Ophthalmic imaging in alert children was attempted using OPTOS Optomap ultra-widefield red green and fundus autofluorescence (FAF) programmes, and/or spectral-domain optical coherence tomography (OCT) using the Heidelberg Spectralis OCT, Monaco and Flex full or fast scan programmes and/or hand-held Bioptigen Envisu. In addition, one child was imaged using the Bioptigen whilst under GA for an MRI. OCT images were graded using the Weill Cornell Ophthalmic Severity Score [25]. The retinal central subfoveal thickness (CST) of the highest quality OCT axial fovea scan was measured from the inner limiting membrane to Bruch’s membrane using the 1:1 µm image view section and callipers as recommended for accuracy by Heidelberg. Visual acuity was assessed using standard clinical tests appropriate for ability and if this was not possible fix and follow behaviour was noted.

This study was registered with the Great Ormond Street Hospital for Children GOSH Clinical Governance and Audit office #2864.

## Results

Twenty-two patients with CLN2, including three siblings pairs, were reviewed. Eighteen patients had at least one of the common mutations C509-1G>C or c.622C>T [33]. Most frequent in this case series was c.509-1G>C in 14 children, five of whom were homozygous. Seventeen children exhibited classical LINCL, with a mean age at diagnosis of 3y10 m (46 m, range 15–51 m), including the pre-symptomatic diagnosis of a younger sibling at 15 m. Five children had atypical disease with a later mean age of diagnosis of 10y3m (123 m, range 95–161 m). A summary of age, genotype and data outcome including the CLN2 clinical rating motor language (ML) score [23] are detailed in Table 1.

**Table 1:** Tabulated result summary.

ID	Age (months)			Nucleotide change		ERG grade (amp/fifth centile)			OCT CST µm		WCBS grade		Vision	CLN2 scale	Flash VEP 3/s		30 Hz	PREV VEP	
	Diag	ERT	Test	Mutation 1	Mutation 2	RE + LE/2	RE	LE	RE	LE	OCT	OPTOS	Vision	ML	#1	#2	VEP	Waveform	Chk width
1	49	52	52	c.622C>T	c.1094G>A	1.0	1.0	1.0				1	F + F	4	y		y	Early 84 ms	25
2	46	47	47	c.622C>T	c.509-1G>C	1.2	0.9	1.4				2	F + F	4	y	PPR	y	Early 87 ms	25
2#2			63	c.622C>T	c.509-1G>C	0.7	0.7	0.7						4	PPR		y	81 ms	50
3	53	58	58	c.622C>T	c.1678–1679 del	0.8	0.6	0.9					F + F	2	PPR	PPR	y	122 ms	25
3#2			68	c.622C>T	c.1678–1679 del	0.5	0.5	0.5						2	PPR		Absent	bifid 74 ms 123 ms	25
13	53	54	54	c509-1G>C	c17 + 1del likely path	1.2	1.3	1.0				4	F + F	4	PPR		y	bifid 72 ms 126 ms	12.5
15	54	55	55	c.509-1G>C	c.509-1G>C	1.6	1.7	1.4						4	PPR		y	102 ms	6.25
15#2			67	c.509-1G>C	c.509-1G>C	0.8	1.3	0.6	150b		2			2	PPR		y	128 ms	12.5
20	49	53	117	c.509-1G>C	c.509-1G>C	nd	nd	nd	50b	30b	5			0	nd		nd	nd	
6	53	55	84	c.509-1G>C	c.509-1G>C	0.9	0.9	0.9						1	PPR	PPR	y	109 ms	25
6#2			92	c.509-1G>C	c.509-1G>C	0.2	0.2	0.3	50b	nd	5			0	PPR		y	Absent	>400
7	49	70	108	c.509-1G>C	c.509-1G>C	0.0	0.0	0.0	73	nd	5		no F + F	0	Absent		Absent	nd	
8	27	47	82	c.509-1G>C	c.509-1G>C	0.2	0.3	0.1	82b	nd	5	5	Fix	4	?PPR		Absent	92 ms	100
14	48	49	50	c.509-1G>C	c1525C>T	1.0	nd	1.0						4	nd		nd	LE ERG only	
9	50	52	84	c.1052C>T	c.1052C>T	0.0	0.0	0.0	52b	40b	5	5	3.8 CPD	2	PPR		Absent	118 ms	200
19	45	46	75	c.509-1G>C	c.622C>T	0.0	0.0	0.0	95b	nd	5		Reacts to light	4	nd		nd	nd	
18	47	52	65	c.1266G>C	c.1266G>C	Declined flash stimulation							2.9 CPD	4	nd		nd	128 ms	25
18#2			131	c.1266G>C	c.1266G>C	0.0	0.0	0.0	80b				NPL	1	Absent		Absent	Absent	>400
17	41	43	104	c.754_757	c.1094G>A	Declined flash stimulation			73	60	3/4		F + F	4	nd		nd	Early 87 ms	50
4	55	56	93	c89+5G>A	c.509-1G>C	2.6	2.9	2.2					F + F	1	PPR	PPR	nd	Early 65 ms	6.25
4#2			104	c89+5G>A	c.509-1G>C	1.7	1.5	1.8	188	192	2/3			0	PPR		y	Early 65 ms	25
21	15	21	65	c89+5G>A	c.509-1G>C	2.2	2.2	2.2	210b	205b	1		F + F	6	PPR		y	Early 88 ms	6.25
22	51	52	65	c379C>T	c.509-1G>C	1.2	1.4	1.0	160b	170b	2	1	F + F	2	PPR		y	Early 60 ms	25
10^a^	95	101	105	c.622C>T	c511G>C	2.8	2.7	2.9				1		4	y		y	Early 77 ms	12.5
11^a^	129	130	134	c.1340G>A	C509-1G>C	2.2	2.3	2.2	223	224	2	4		4	PPR		y	bifid 97 ms 144 ms	6.25
12^a^	158	159	164	c.1340G>A	C509-1G>C	2.0	1.9	2.1	180	189	1	3		4	y		y	139 ms	6.25
5^a^	161	180	209	c.89+5G>C	c.1340G>A	1.7	1.6	1.7	147	145	2/3	2	0.2 LogMAR	4	y	PPR	y	108 ms	6.25
5^a^#2			225	c.89+5G>C	c.1340G>A	1.6	1.6	1.7	131	135	2/3			4	PPR		y	Early 99 ms	6.25
16^a,b^	72	NA	73	c.887 G>A	c.887G>A	2.4	2.6	2.1	197	206	1	1	0.02 LogMAR	6	?PPR	PPR	y	113 ms	6.25
16^a^#2			120	c.887G>A	c.887G>A	1.9	1.9	1.9	197	198	1			6	PPR		y	116 ms	6.25

*Age (months)*: Ages in months are given for diagnosis (diag), first enzyme replacement therapy (ERT) and electrophysiology test (test).

*Nucleotide change*: common mutations c.509-1G>C and c.622C>T are shaded grey.

ERG grade is scored by dividing the measured amplitude for each response by the fifth centile reference limit. The indices for each response are then averaged. Broadly, 1 is borderline and below one is subnormal.

*OCT CST:* is the central subfoveal retinal thickness, b indicates Bioptigen, others Spectralis, nd not done.

WBCS is the Weill Cornell Batten Score (Kovacs et al. [26]) formerly ophthalmic severity score (Orlin et al. [25]). *Range 1–5*: 1 is normal and 5 is generalised retinal atrophy.

CLN2 scale is the motor language scale associated with the date of each test. Range 0–6: 0 is a severe disability and 6 is normal.

*Flash VEP*: #1 first test, #2 second test, PPR photo paroxysmal response, abn abnormal, y is present.

*PREV VEP*: ms is the latency of the major positive peaks. P100 reference limit with 95% CI is 90–112 ms (mean 101 ms).

*Chk width*: is the smallest check side length in minutes of an arc that produced a response with both eyes open (range 400′–6.25′).

Patient ID. ^a^Are patients with atypical forms of CLN2. ^b^Is not on ERT because of non-progressive disease at this stage. Sibling pairs are 7 & 8, 11 & 12, 4 & 21.

Flash ERG recordings were attained in 20/22 patients. Two patients declined flash testing, one on one occasion, because of concerns about photosensitive epilepsy. Testing was unsuccessful in one patient because of agitation. Nine of twenty recorded patients had normal flash ERGs at the last test, four had absent ERGs. The amplitudes and time to peaks of measurable ERGs are tabulated in Appendix 1 and graphically displayed for right and left eyes with the lab reference values for each stimulus in Appendix 2. The ratio of b-wave to a-wave mixed rod-cone amplitudes did not show any electronegative ERGs.

**Fig. 1:**
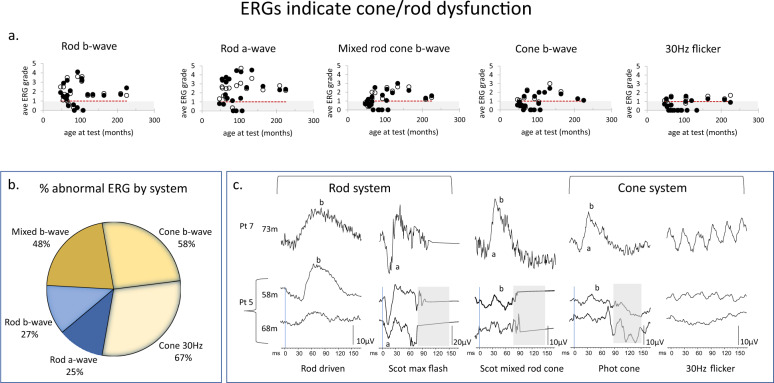
ERG data from children with CLN2 disease. **a** Scatter plot of graded ERGs from RE and LE as a function of age for each of the five ERG responses. The grey region indicates subnormal amplitudes. **b** Venn diagram highlights the greater proportion of abnormal cone system ERGs 58–67% compared to rod system 25–27%. **c** Examples of each of the five ERG waveforms from two patients. Pt 7 (hom c.887 G>A) shows an attenuated atypical form of CLN2. Skin ERGs are noisy, (muscle artefact), but were within the normal reference range for amplitude and waveform at 73 m and follow-up at 120 m. Pt 5 (c.622 C>T, c.1678–1679 del) in contrast at first recording aged 58 m had subnormal cone ERGs but normal rod ERGs. At follow-up 10 months later there is loss of a detectable cone 30 Hz flicker ERG and the rod system ERGs begin to lose amplitude.

To compare these ERG data with other studies that have used different recording methods the ERG amplitudes were graded by dividing the measured value by the fifth centile reference limit for each stimulus. These are plotted for each eye and each stimulus as a function of age in Fig. 1a. The array of individual ERGs above 1 shows a greater number of rod system ERGs with normal amplitude compared to cone system ERGs. The Venn diagram shows the relative proportions of abnormal ERGs for each stimulus, emphasising relatively greater cone system dysfunction Fig. 1b. The paired right eye (RE) and left eye (LE) ERG amplitude values did not differ significantly for each stimulus at the 0.001 level, Wilcoxon ranked signed test. The output for each statistical test is tabulated in Appendix 3. Fig. 1c. shows example ERG waveforms from two patients. The top row is from a patient aged 6y1m, (73 m) who had normal amplitude ERGs. Below is an example of a patient with serial ERG recordings that diminished quickly over a 10 month inter test period; cone ERGs reduced more than rod system ERGs.

**Fig. 2:**
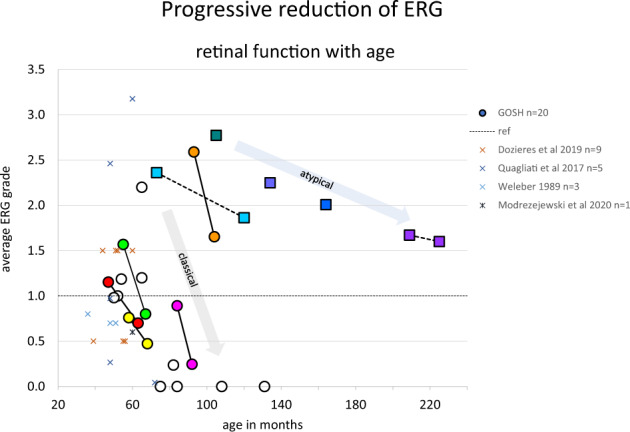
Averaged ERG grades, combined from RE and LE for all stimuli to provide a single comparable index for each patient, are displayed as a function of age. The dotted reference line at 1 indicates borderline ERGs, values below this are subnormal amplitude. Circles indicate patients with classical CLN2 disease, boxes indicate those with atypical disease. Serial ERG data from seven patients are linked by lines to show the trajectory of ERG amplitude loss. This grade enabled comparison with available published ERG data produced by different techniques. These data are shown as X symbols. Weleber = 3 cases with traces described as proportions of mean, no genotype; Quagliati = 5 cases with values and fifth centile reference data against which the amplitudes were normalised, no genotype; Modrzejewski = 1 case with published traces graded cf Control traces, no genotype; Dozieres = 9 genetically diagnosed cases, ERGs tabulated with a binary classification abnormal or normal which was translated as 0.5 for abnormal and 1.5 for normal for purposes of graphing vs. age.

The ERG grades from all stimuli for each eye were averaged to provide a single ERG index for each patient and these are plotted for 20 patients as a function of age in Fig. 2. Seven of the 20 patients had serial ERG data. The lines joining data points show a loss of amplitude over time for all individual patients with serial recordings. The rate of amplitude loss approximates to 0.6 ERG grade /annum in the classical group but is more gradual in two patients with non-classical forms of CLN2 disease. The ERG data from GOSH vs age are compared with available published ERG data replotted from four series of cases described as LINCL [27, 34, 35], with genotype confirmed in one study [36].

**Fig. 3:**
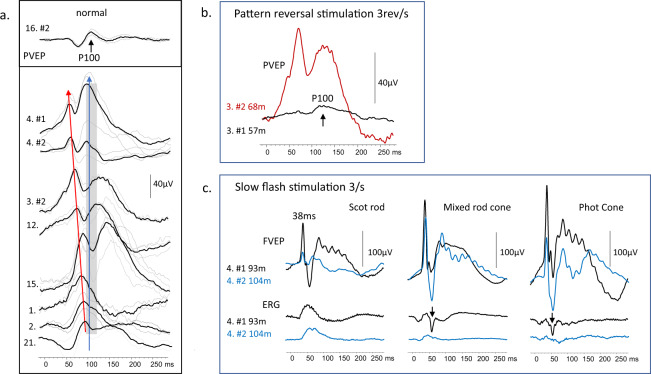
Examples of pattern and flash VEPs and flash ERGs recorded from patients with CLN2 disease. **a** A column of pattern reversal VEPs recorded from Oz to mf reference to 50′ check widths are arrayed to display the early atypical positive-negative-positive configuration highlighted with a red arrow compared to the P100 (black arrow on the top trace). The grey vertical panel shows the normal latency reference limits with 95% CI. Individual patients ID next to each trace #1 and #2 denotes first and second recording. **b** Serial pattern VEPs to 50′ check widths for patient 3 are shown. At the first recording aged 57 m, a single positive pattern VEP waveform is noted with a recognisable P100 (black trace). The waveform is typical compared to a normal waveform shown in Fig. 4a. At the follow-up recording, 9 m later the reversal VEP is overwhelmed by a giant pattern-driven paroxysmal EEG activity **c** The photo-paroxsymal response (PPR) to flash stimulation can cause a giant spike and wave from the occipital electrodes to slow flash presentations 3/s. An early giant flash VEP due to the intrusion of PPR is shown from patient 4 aged 93 m for three different flash stimuli all presented 3/s. The most pronounced largest PPR is seen during cone system stimulation. The PPR is sufficient to confound the simultaneously recorded cone and mixed rod-cone ERGs. They have a prominent negative-going artefact (arrowed), either reflected from the PPR in the frontal region and/or with myoclonic periocular reaction. This gives the cone ERG an appearance of two peaks. The rod system b-wave is minimally affected. (The lower lid cheek skin ERG electrode was referred to as an outer canthus reference). At a follow-up recording 11 months later (Blue traces), the PPR is diminished and less complex. It interferes less with the skin ERGs which are evident and borderline normal. NB the flash VEP display scale is 100 µV the skin ERGs are shown on 10 µV scale.

Pattern reversal VEPs recordings took place with 19 patients, examples produced by the ISCEV standard large check width (50′) are shown in Fig. 3a. The PVEP had an atypical waveform in almost 50% of patients. A single positive peak at 100 ms is expected. Our latency reference range for 50′ with 90% CI reference limits is between 90 and 112 ms (mean 101 ms). Children with CLN2 showed unusually early (52–88 ms), occasionally abnormally large, positive peaks. This atypical activity is time-locked to the pattern stimulus. This was a striking change for patient 3, who aged 58 m (4.8 y) showed a typical morphology PVEP, but within 11 months had developed an abnormally early and giant response whilst the ERG deteriorated (Fig. 3b). Flash VEPs also showed atypical features in 12/16 children (75%). The example shows the largest intrusion was associated with cone system flash stimulation. This confounded the simultaneously recorded ERGs when the patient was 7y9m, (93 m). The amplitude of the atypical activity reduced in an 11 month period, Fig. 3c.

**Fig. 4:**
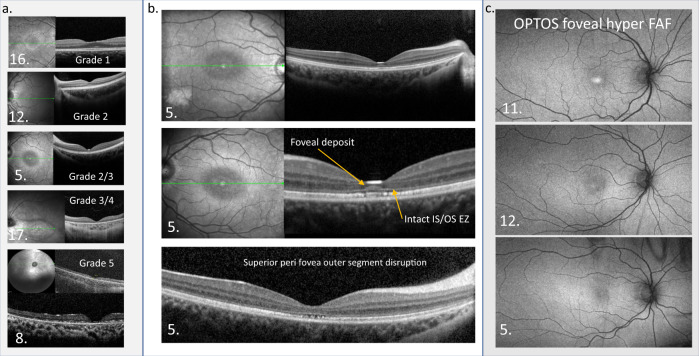
Macular OCT and OPTOS FAF images. **a** Examples of macular OCTs taken from the case series to show the structural evolution and grades of the maculopathy. **b** OCTs from patient 5 in the central panel highlight the atypical accumulation adjacent to the external limiting membrane. The perifoveal disruption of outer segments is also shown below. This did not have a functional consequence on VA or PERG. **c** OPTOS FAF images are arrayed from 3 patients who have a normal central thickness at the time of imaging but show foveal hyper FAF, suggesting this is an early sign of retinal dysfunction in CLN2.

OCT reference images have been arrayed to highlight the progression of foveal retinal thinning to generalised retinal atrophy in Fig. 4a with the Weill Cornell Battens grade [25, 26]. The central panel, Fig. 4b, shows an unusual ovoid, aggregation abutting the outer limiting membrane at the fovea of each eye in patient 5, together with more perifoveal outer segment disruption. The PERGs from the 15° field was not affected. In Fig. 4c OPTOS FAF images from 3 patients showing foveal hyper FAF when ERGs are within normal reference range are arrayed.

**Fig. 5:**
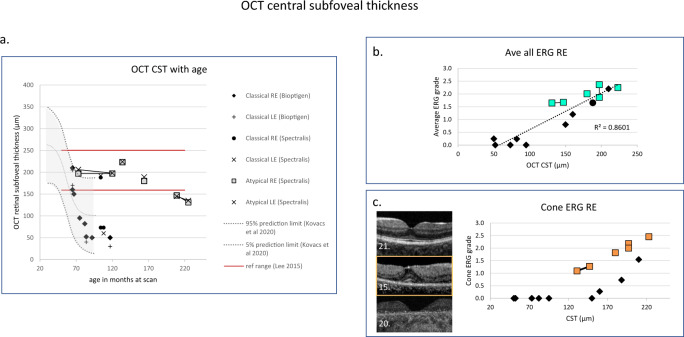
Changes in OCT central subfoveal thickness with age and ERG score. **a** OCT CSTs are plotted with age and compared with the right eye prediction interval data redrawn from Kovacs et al. 2020 [26]. GOSH patients with the classical disease are identified by filled symbols, those with the atypical disease with boxes. The red horizontal reference data lines represent ±3 SD of the CST expected for this age range published by Lee et al. 2015 [56]. The solid symbols are from patients with classical CLN2 disease and grey box symbols indicate patients with atypical disease. **b** The right eye OCT central retinal thickness is correlated with the average ERG score from the right eye. Shown with a linear regression line. (The Kendall correlation was 0.71, significant *p* 0.003). **c** The OCT CST plotted with cone ERG data shows a dichotomy of structure and function. Those with classical CLN2 disease who retain CST (filled symbols) have reduced retinal function. OCT images from 3 patients with classical CLN2 disease are shown in the panel. The middle macula image (OCT Bioptigen) in the panel (patient 15.) has retained the CST, but there is a marked drop out of the ellipsoid zone and the ERG score is subnormal.

Optomap images were captured in 11 patients and OCTs achieved in 16 alert patients with serial measures in 2 patients, [7 patients with Spectralis and 9 patients with Bioptigen OCT]. The RE and LE CST values correspond within 20 microns, and the paired RE and LE data did not differ significantly at the p 0.001 level, Wilcoxon signed-rank test. The CST of these 16 patients were plotted against age in Fig. 5a and also compared to the RE OCT centre thickness prediction intervals modelled by Kovacs et al. from the CST of 16 children measured under anaesthesia, 5 of whom had serial recordings [26]. Although a strong association was found between OCT centre thickness data and average ERG score from all stimuli, Kendall correlation 0.71, *p* 0.0003 (Fig. 5b), for similar CST the cone ERG data show a greater functional loss in patients with classical disease than the atypical group (Fig. 5c).

## Discussion

This case series of ERGs from twenty patients show that CLN2 retinopathy is functionally a symmetrical cone-rod dystrophy, with early structural OCT signs of maculopathy. The OCT CST, from the fovea where the cone concentration is highest (200,000), correlates with the average ERG score that sums activity from the majority of cones in the more peripheral retina (6.5 million), [37, 38]. This suggests a wide dropout of cones across the retina. The ERG b-wave time to peaks tends to remain within reference range until amplitudes are subnormally confirming a loss of cone cells rather than dysfunction (Appendix 2). This implies cone metabolism is specifically vulnerable to a deficit of TTP1; supported by an electron microscopy report [39] and the few CLN2 ERG case reports [27, 34, 35]. Importantly for similar CST measurements patients with classical CLN2 disease show a greater loss of cone ERG than those with atypical disease. This shows that retinal cone dropout in classical CLN2 disease can precede and overtake signs of structural CST thinning at the fovea.

Maintenance of CST in some cases may relate to bilateral ovoid accumulations above the outer limiting membrane at the fovea, seen most clearly in the oldest patient. Interestingly, the Dachshund model of CLN2 disease is accompanied by an accumulation of autofluorescent storage material along the outer limiting membrane as well as in the ganglion cells [40]. The accumulation did not auto fluoresce with OPTOS FAF imaging, though a younger sibling pair with preserved central retinal thickness did show some early signs of foveal hyper FAF.

The lack of a frank electronegative ERG waveform highlights the CLN2 retinopathy in these children is not a primary inner retinal dysfunction, (Fig. 1 patient 5), noted also by Weleber [27]. This contrasts with the canine TPP1 null variant, where retinal degeneration is described as a progressive loss of inner retinal function, affecting the rod system more profoundly than the cone system [40, 41]. In particular, the canine ERG is profoundly electronegative, with a-wave amplitudes declining later in the disease progression [40, 41]. The canine model more closely resembles the ERG of human CLN1 and CLN3 disease, [5, 28, 41]. Canine models of other human cone disease show reduced cone ERGs and preservation of rods ERGs [42], and the reason for this disparity in CLN2 is unclear, but it is an important consideration when piloting human eye treatments based on the canine model.

A more severe CLN2 retinopathy appears associated with c.509-1G>C, but this was also the most frequent in our series. The youngest patient with a subnormal ERG is 4y10m (58 m), whilst patients with no detectable ERG and profound retinal dysfunction are aged 6.25–8.75 y (75–105 m) broadly aligning with the trajectory of visual loss over 3 y described by Steinfield [21]. Our series highlights a dichotomy of more rapid ERG amplitude loss in classical CLN2 and a gradual loss in atypical disease. This phenotypic variability may reflect residual TPP1 production and other genetic modifiers of disease severity [10, 36, 43].

The lack of visual response from children with CLN2 reflects post-retinal neurodegeneration of the visual pathway and occipital cortex as well as retinal dysfunction. Visual pathway function is reflected in the VEP extracted from the electroencephalogram (EEG). In CLN2 the EEG is often the first test to detect irregular activity as slowing of background activity, and epileptiform abnormalities in posterior regions [44]. In contrast to other studies that have reported absent or delayed flash VEPs [34, 36, 45, 46] we were able to record VEPs in the majority 16/18 (89%) of patients, but 12/16 (75%) had a confounding photo paroxysmal response (PPR). Two patients developed PPR sixteen months after their first flash VEP recording, another showed a reduction in PPR between 93 and 104 m (7.8–8.7 y) (Fig. 3c), suggesting it is associated with a particular stage of neurodegeneration. Large polyspikes in the EEG associated with flash stimulation at slow rates have been described as a characteristic of LINCL [47] and other studies have reported PPR in 9/13 (69%) CLN2 patients at a median of 48 m [44] and estimated 60% of CLN2 patients show PPR [48]. It has been suggested that the deficit of TPP1 may alter the metabolism of cells responsible for electrogenesis [44]. As PPR reflects cortical hyperexcitability to slow afferent stimulation the faster, 30/s flicker VEP may offer an alternative way of monitoring visual pathway function.

The flash VEP waveform shows wide inter-individual variability and the pattern reversal VEP waveform is preferred clinically because it has a relatively constant single positive peak throughout life. The reversal VEP is a strong index of macular pathway function. Pattern reversal VEPs in 2/18 patients showed a bifid waveform which is associated with a central scotoma, caused by maculopathy or macular pathway dysfunction such as optic atrophy. Although a majority of patients (16/18) showed evidence of PVEPs to check widths of 50′ or smaller at the first recording many (10/16, 63%) were atypically early with large amplitudes due to the intrusion of pattern-driven paroxysmal EEG activity. Pattern sensitive or pattern-driven paroxysmal responses can falsely augment and disguise the true PRVEP as seen in Fig. 4. The later appearance of giant VEPs or pattern sensitivity in serial pattern reversal VEPs suggests a change with disease progression, remarked also by Pampiglione [47]. As illustrated in Fig. 3c. the PPR intrusion can even confound the ERG recording. Such abnormal spikes can be seen in published canine ERGs too [40].

The symmetry of retinal disease progression is very important for future therapies if the fellow eye may act as a control. Kovacs et al. [26] reported symmetry of OCT progression in 5 CLN2 patients. We confirm an inter-ocular symmetry of OCT measures at selected time points in ten patients. We additionally show an inter-ocular symmetry of functional ERG measures at a selected time points and in the progression trajectory of ERGs in seven patients.

The limitations of this case series are in common with retrospective studies but accentuated in studies of rare diseases when children are very challenging to examine. Data were acquired after the children had completed their fortnightly TPPI ERT infusions. There are missing data when compliance became insufficient for reliable data collection. It was physically difficult to sustain the best position with a desktop mounted system. An arm-mounted OCT was used with some success with the fast acquisition programme, but the large machinery can be nudged easily by the children. The Bioptigen is a smaller handheld option. Studies comparing axial CST measurements from the same instruments show excellent inter-observer agreement, but different instruments require a correction factor to translate data from one platform to another. For our study ×1.025 is suggested from model eye evaluations to translate from handheld Bioptigen Envisu™ to Heidelberg Spectralis™ [49]. The more severely affected children with the thinnest CST tended to be imaged with the Bioptogen. For this cross-sectional study, there is little impact to our conclusions, with a maximum difference of ~8 µm, and no translational scaling was used, but this could be pertinent for future treatment trials. We noted an operator bias from imaging the RE first. Fewer LEs were imaged successfully as alert children lost tolerance. Although more complete data may be gathered from children under anaesthesia, particularly OCT, the neurodevelopmental effects of repetitive anaesthesia are uncertain [50, 51], and anaesthetic agents can alter the ERG, in particular reducing ERG b-wave amplitudes and delaying the b-wave time to peaks, [52, 53].

As noted by others the clinical fundus is found often to be unremarkable in CLN2 retinopathy [34, 36]. The OPTOS FAF images were helpful, identifying localised foveal hyperFAF, and the colour image helped the grading by highlighting areas of atrophy. We did not observe the general increase and decrease of FAF with disease progression reported in a mixed group of NCL patients [54]. Offline analysis may be required for this. Also, the FAF findings may have been different with the 488 nm blue FAF of cSLO Heidelberg Spectralis™, compared to the 532 nm green FAF OPTOS Optomap, which the children tolerated. The ophthalmic severity grade did not incorporate FAF images, [25].

Our large case series of children with CLN2 demonstrate a wide phenotypic variability with age, but the classical disease retinopathy fits broadly within the CST OCT prediction interval suggested by Kovacs et al. [26]. Those with classic CLN2 disease also show similar rapid rates of ERG loss from different starting ages. Different ages of progression onset also can be seen in better than predicted pre-treatment ML scores [24, 55]. The symmetry of CLN2 retinopathy lends support to using the fellow eye as a control in future ocular treatment studies. The ERG data in this case series show CLN2 retinopathy is an early cone/rod disease which is an important distinction from the dachshund TPP1-null canine model that shows an electronegative rod/cone phenotype. It also shows functional loss can precede structural CST thinning in classical CLN2 disease.

### Summary

#### What was known before

- Structurally, OCT data show Batten disease CLN2 retinopathy starts as a maculopathy and progresses to involve cones and rods.
- Clinical assessment of children with CLN2 is challenging and grading scales may help.

#### What this study adds

- Functionally, ERG data show CLN2 retinopathy is cone-rod dystrophy.
- Children with CLN2 retinopathy do not show the profoundly electronegative ERGs described in the canine CLN2 model or in human CLN1 and CLN3 phenotypes.
- ERG changes can precede central subfoveal thickness change in classical CLN2 disease.
- There is a phenotypic variation of CLN2 retinopathy in classical and non-classical diseases and the age when normal ERGs are recordable.

## Supplementary information

Appendix 1 - Measured ERG values

Appendix 2 - ERG data plotted wrt reference ranges

Appendix 3 - Statistical tables
