# Correction: An ERG and OCT study of neuronal ceroid lipofuscinosis CLN2 Battens retinopathy

**DOI:** 10.1038/s41433-021-01720-w

**Published:** 2021-08-12

**Authors:** Dorothy A. Thompson, Siân E. Handley, Robert H. Henderson, Oliver R. Marmoy, Paul Gissen

**Affiliations:** 1 Clinical and Academic Department of Ophthalmology, Great Ormond Street Hospital for Children, London, UK; 2 UCL Great Ormond Street Institute of Child Health, London, UK; 3 Genetics and Genomic Medicine, Great Ormond Street Institute of Child Health, University College London, London, UK; 4 NIHR Great Ormond Street Hospital Biomedical Research Centre, University College London, London, UK

**Keywords:** Retina, Neuroscience

Correction to: *Eye*:10.1038/s41433-021-01594-y

Unfortunately, the spelling of the author’s name was incorrect. The correct name should read Paul Gissen.

The original article has been corrected.

