# Supplementary figures and images for: An ERG and OCT study of neuronal ceroid lipofuscinosis CLN2 Battens retinopathy
Source: Eye (Lond). 2021 Jul 16;35(9):2438–48. doi: 10.1038/s41433-021-01594-y (PMC8377094; doi:10.1038/s41433-021-01594-y)

## Slide 1
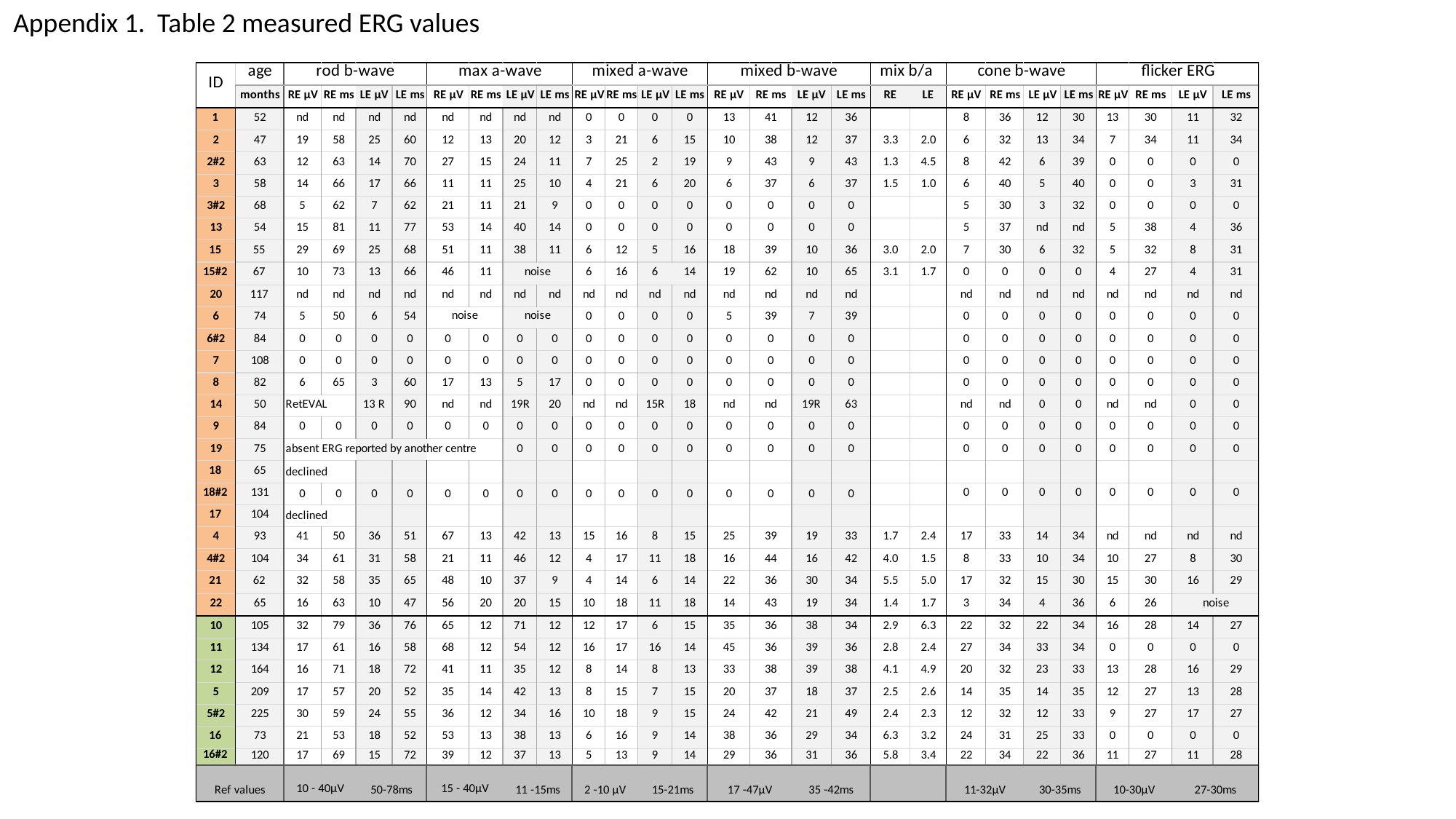

Appendix 1. Table 2 measured ERG values

Supplement: Supplementary file 1 — Appendix 1 - Measured ERG values [file 41433_2021_1594_MOESM1_ESM.pptx]
