# Supplementary material for: An ERG and OCT study of neuronal ceroid lipofuscinosis CLN2 Battens retinopathy
Source: Eye (Lond). 2021 Jul 16;35(9):2438–48. doi: 10.1038/s41433-021-01594-y (PMC8377094; doi:10.1038/s41433-021-01594-y)
Supplement: Supplementary file 2 — Appendix 2 - ERG data plotted wrt reference ranges [file 41433_2021_1594_MOESM2_ESM.pptx]

## Slide 1
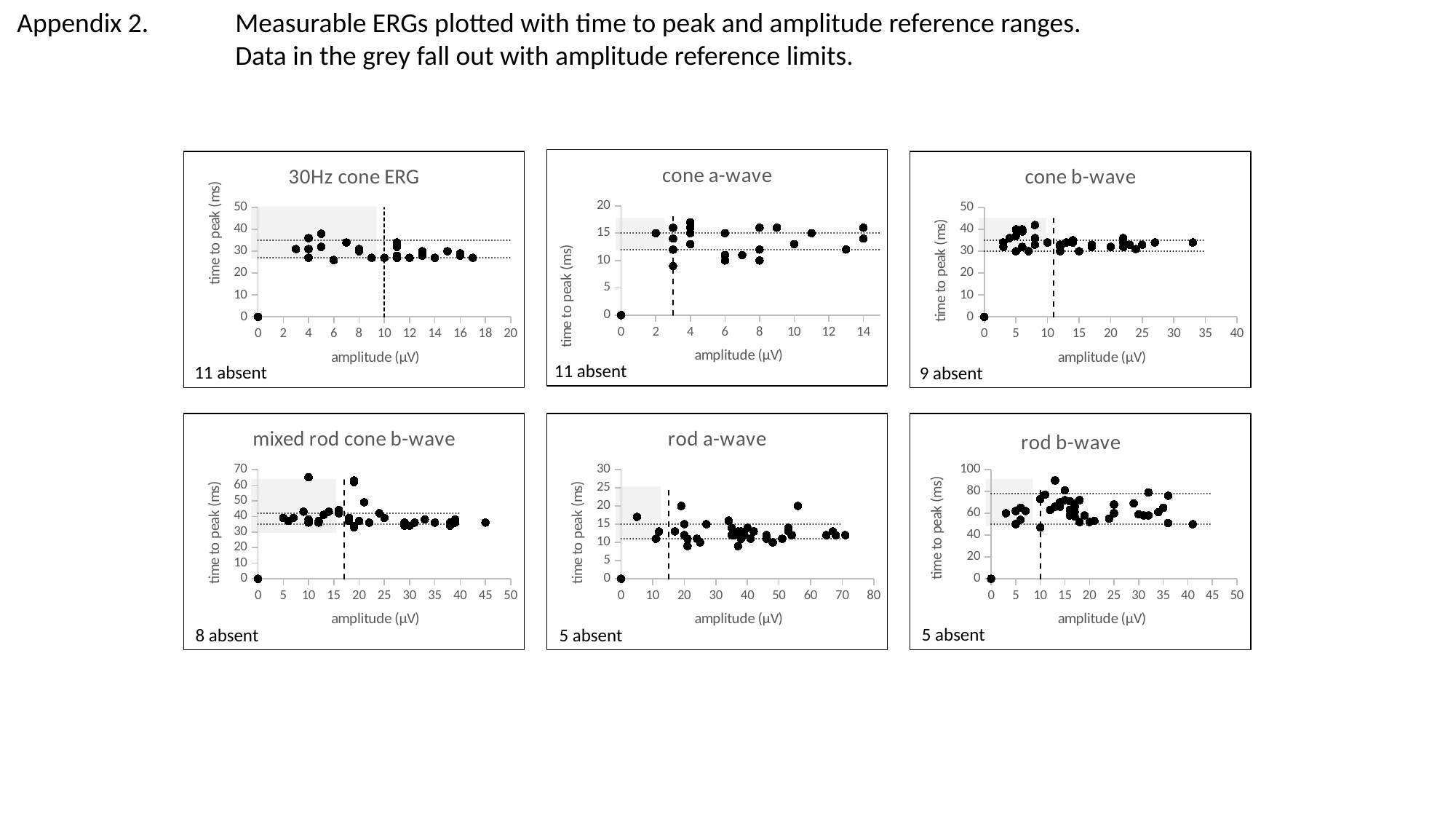

Appendix 2. 	Measurable ERGs plotted with time to peak and amplitude reference ranges.
		Data in the grey fall out with amplitude reference limits.
### Chart: cone a-wave
| Category | | | | | |
|---|---|---|---|---|---|
### Chart: 30Hz cone ERG
| Category | | | | | |
|---|---|---|---|---|---|
### Chart: cone b-wave
| Category | | | | | |
|---|---|---|---|---|---|
11 absent
11 absent
9 absent
### Chart: mixed rod cone b-wave
| Category | | | | | |
|---|---|---|---|---|---|
### Chart: rod a-wave
| Category | | | | | |
|---|---|---|---|---|---|
### Chart: rod b-wave
| Category | | | | | |
|---|---|---|---|---|---|
5 absent
5 absent
8 absent
