# Supplementary material for: An ERG and OCT study of neuronal ceroid lipofuscinosis CLN2 Battens retinopathy
Source: Eye (Lond). 2021 Jul 16;35(9):2438–48. doi: 10.1038/s41433-021-01594-y (PMC8377094; doi:10.1038/s41433-021-01594-y)
Supplement: Supplementary file 3 — Appendix 3 - Statistical tables [file 41433_2021_1594_MOESM3_ESM.pptx]

## Slide 1
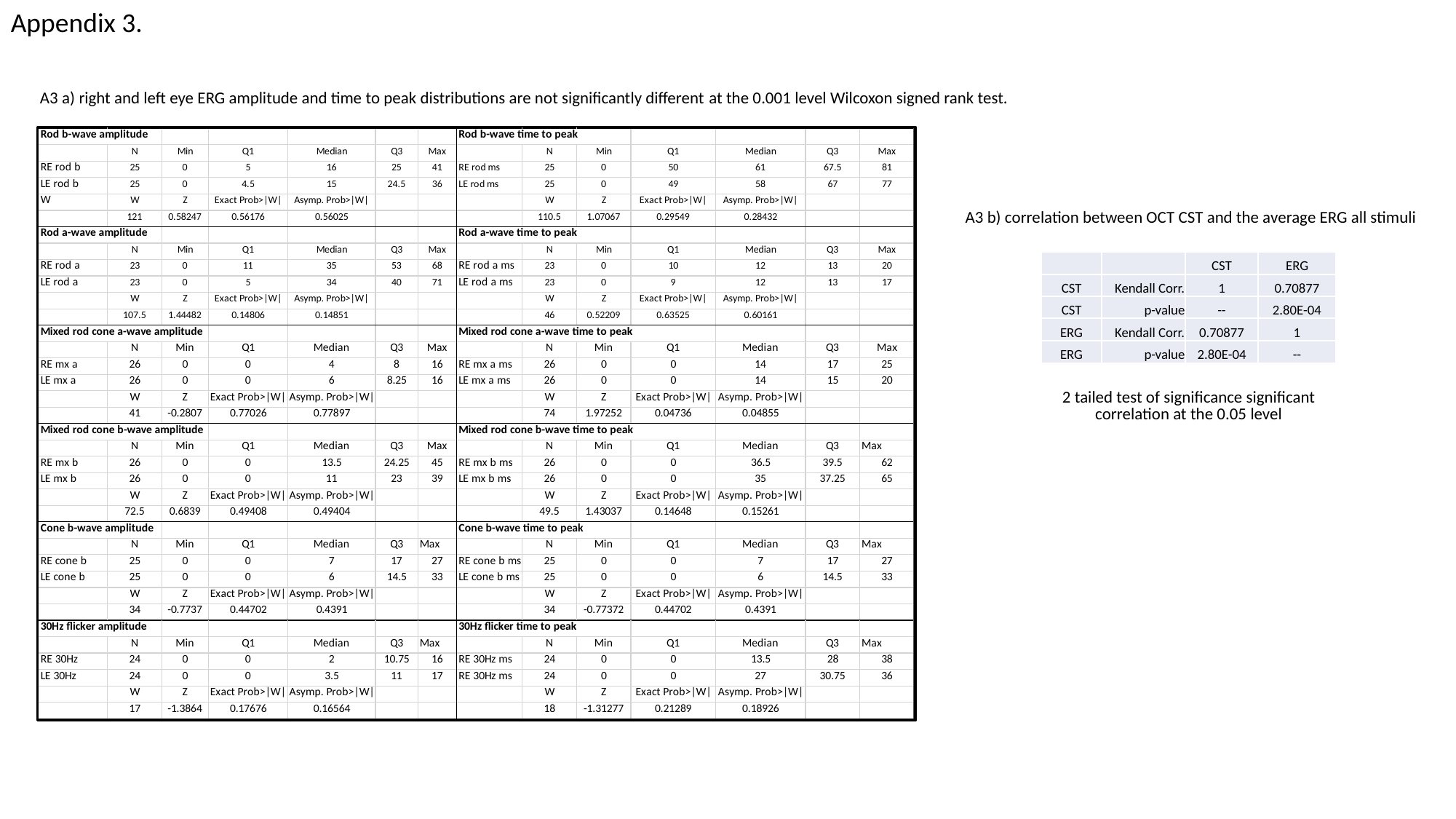

Appendix 3.
A3 a) right and left eye ERG amplitude and time to peak distributions are not significantly different at the 0.001 level Wilcoxon signed rank test.
A3 b) correlation between OCT CST and the average ERG all stimuli
| | | CST | ERG |
| --- | --- | --- | --- |
| CST | Kendall Corr. | 1 | 0.70877 |
| CST | p-value | -- | 2.80E-04 |
| ERG | Kendall Corr. | 0.70877 | 1 |
| ERG | p-value | 2.80E-04 | -- |
| 2 tailed test of significance significant correlation at the 0.05 level | | | |
